# Corydalis Tuber Extract Alleviates Atopic Dermatitis: Transcriptomics-Based Mechanism Prediction and In Vitro/In Vivo Studies

**DOI:** 10.3390/ijms26031291

**Published:** 2025-02-03

**Authors:** Seong-Eun Jin, Chang-Seob Seo, Woo-Young Jeon, Yong-Jin Oh, Hyeun-Kyoo Shin, Hyekyung Ha

**Affiliations:** 1 KM Science Research Division, Korea Institute of Oriental Medicine, Daejeon 34054, Republic of Korea; noellajin@kiom.re.kr (S.-E.J.); csseo0914@kiom.re.kr (C.-S.S.); yjoh@kiom.re.kr (Y.-J.O.); hkshin@kiom.re.kr (H.-K.S.); 2 KM Convergence Research Division, Korea Institute of Oriental Medicine, Daejeon 34054, Republic of Korea; ssamggun85@kiom.re.kr; 3 College of Pharmacy, Keimyung University, 1095 Dalgubeol-daero, Dalseo-gu, Daegu 42601, Republic of Korea

**Keywords:** atopic dermatitis, inflammation, Corydalis Tuber, transcriptome analysis

## Abstract

Atopic dermatitis (AD) is a common inflammatory skin disease characterized by recurrent eczema and chronic itching, affecting a significant portion of the global population. This study investigated the effects of Corydalis Tuber 70% ethanol extract (CTE) on tumor necrosis factor-α- and interferon-γ (TI)-stimulated human keratinocytes (HaCaT) and a house dust mite-induced AD mouse model, elucidating its mechanism via transcriptome analysis. A total of 13 compounds, including columbamine, corydaline, dehydrocorydaline, and glaucine, were identified in CTE using ultra performance liquid chromatography-tandem mass spectrometry. CTE downregulated pathways related to cytokine signaling and chemokine receptors in TI-stimulated HaCaT cells. It significantly inhibited C-C motif chemokine ligand (CCL)5, CCL17, and CCL22 levels by blocking the Janus kinase-signal transducers and activators of transcription and nuclear factor kappa-light-chain-enhancer of activated B cells pathways. In the AD mouse model, topical CTE significantly decreased dermatitis scores, epidermal thickening, and inflammatory cell infiltration. Plasma levels of histamine, immunoglobulin E, CCL17, CCL22, corticosterone, and cortisol were reduced. Lesions showed decreased thymic stromal lymphopoietin, CD4^+^ T cells, interleukin-4, and intercellular adhesion molecule-1 expression. The findings demonstrate that CTE alleviates AD by modulating inflammatory mediators, cytokines, and chemokines, reducing inflammatory cell infiltration, and alleviating stress-related factors.

## 1. Introduction

Atopic dermatitis (AD) is a prevalent inflammatory skin disease with a considerable global impact, affecting individuals across various age groups [1]. Patients with AD experience severe physical and mental pain, owing to recurrent eczema and chronic itching [2]. Numerous variables are involved in the pathophysiology of AD, including genetic predisposition, skin barrier dysfunction, immune dysregulation, and microbial imbalance [1,3]. T cells stimulated by various allergens and stimuli produce proinflammatory cytokines and chemokines [3,4]. These chemokines work in concert with various adhesion molecules, such as intercellular adhesion molecule-1 (ICAM-1), to recruit pathogenic leukocytes directly into the skin. Thymic stromal lymphopoietin (TSLP), released by keratinocytes, directs dendritic cells to stimulate Th2 cell differentiation. Th2 cell overactivation increases immunoglobulin E (IgE) production, which in turn activates mast cells and exacerbates AD [3].

Topical agents, such as corticosteroids and calcineurin inhibitors, are commonly used to treat AD; however, they may cause side effects, such as skin burning, warmth, redness, and local allergic reactions, and an increased risk of secondary infection due to reduced immunity [1]. Additionally, if these drugs are absorbed systemically, they may cause breathing difficulties, edema, and systemic immunosuppressive effects, which may increase the risk of infection and malignancy. Therefore, research is ongoing to ameliorate AD and develop treatments based on safer natural products [1,5].

Medicinal plants have historically served as therapeutic agents for various diseases, and many natural products have been developed as new pharmacological items based on traditional medicine [6]. The tuber of *Corydalis yanhusuo* W.T. Wang (Papaveraceae), commonly known as Corydalis Tuber, has been used as an analgesic to treat stomachaches and is beneficial in treating inflammatory, hemorheological, and allergic diseases [7,8]. Corydalis Tuber exerts pharmacological effects, including anti-type I–IV allergy, anti-inflammatory, anti-thrombotic, anti-depression, and anti-anxiety properties [7,9,10,11]. Corydalis Tuber contains abundant alkaloids, such as coptisine, berberine, palmatine, canadine, columbamine, corydaline, dehydrocorydaline, and protopine [12,13,14,15]. However, the inhibitory effects and mechanism of action of Corydalis Tuber on AD have not been studied.

In this study, we aimed to predict the mechanism of action of Corydalis Tuber using in vitro transcriptome analysis and suggest its potential application as an AD treatment using in vitro and in vivo experimental models. The anti-AD properties of Corydalis Tuber 70% ethanol extract (CTE) were confirmed by evaluating the reduction of chemokine production in human keratinocytes (HaCaT) and AD improvement by applying CTE to a house dust mite (HDM)-induced AD mouse model.

## 2. Results

### 2.1. Simultaneous Analysis of Compounds in CTE Using Ultra Performance Liquid Chromatography-Tandem Mass Spectrometry (UPLC-MS/MS)

**Figure 1:**
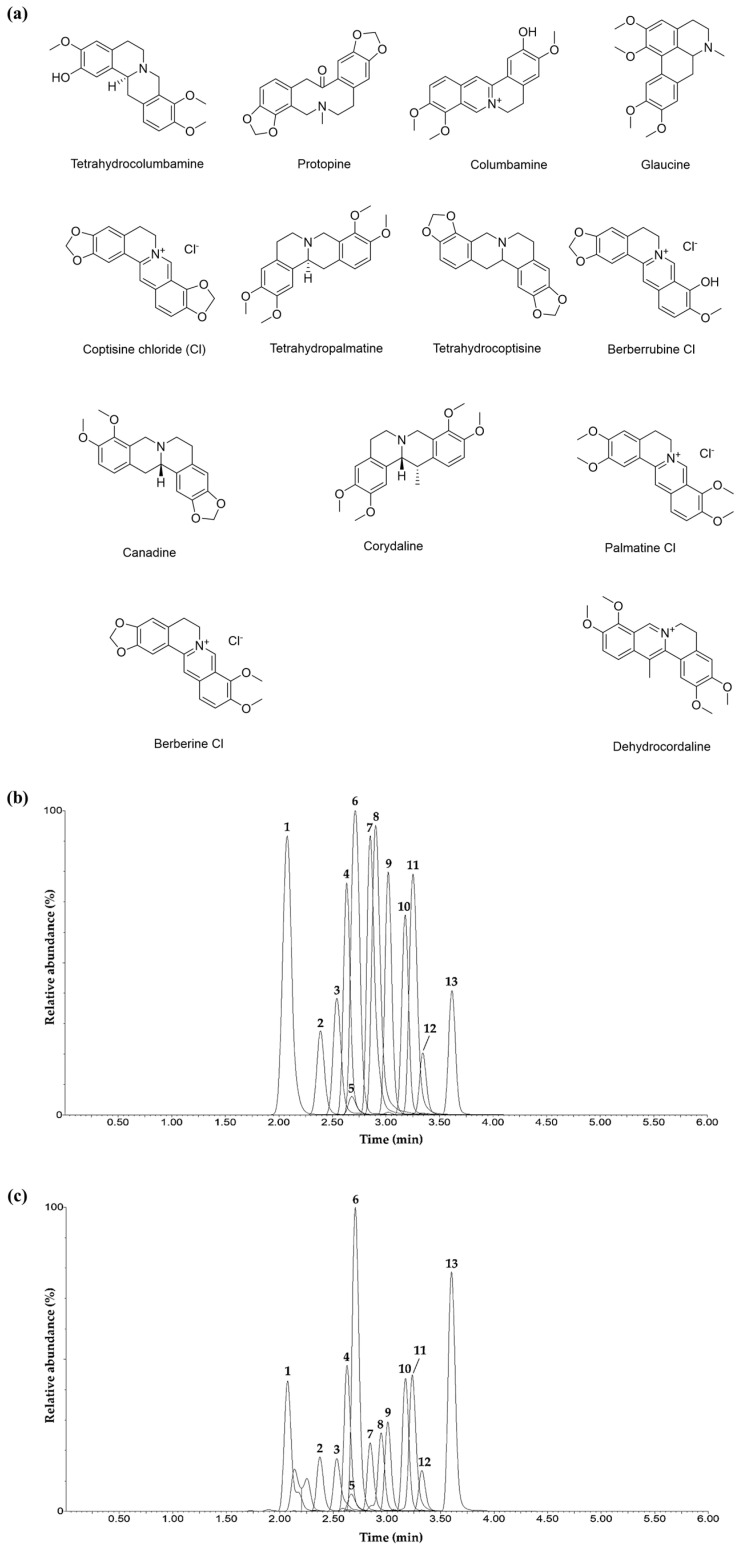
(**a**) Chemical structures of compounds in CTE. Total ion chromatograms of mixed standard solution of compounds (**b**) and 70% methanolic solution of lyophilized CTE (**c**) measured via UPLC-MS/MS MRM in positive ion mode. Tetrahydrocolumbamine (1), protopine (2), columbamine (3), glaucine (4), coptisine Cl (5), tetrahydropalmatine (6), tetrahydrocoptisine (7), berberrubine Cl (8), canadine (9), corydaline (10), palmatine Cl (11), berberine Cl (12), and dehydrocorydaline (13). CTE, Corydalis Tuber 70% ethanol extract; MRM, multiple reaction monitoring; UPLC-MS/MS, ultra performance liquid chromatography-tandem mass spectrometry.

The established UPLC-MS/MS multiple reaction monitoring (MRM) analytical method was applied to simultaneously determine 13 compounds (Figure 1a) in CTE. Tetrahydrocolumbamine, protopine, columbamine, glaucine, coptisine Cl, tetrahydropalmatine, tetrahydrocoptisine, berberrubine Cl, canadine, corydaline, palmatine Cl, berberine Cl, and dehydrocorydaline were detected at 2.07, 2.39, 2.54, 2.64, 2.68, 2.70, 2.85, 2.90, 3.02, 3.18, 3.25, 3.34, and 3.61 min, respectively (Figure 1b,c, Table S3). In MRM mode, seven compounds (tetrahydrocolumbamine, protopine, glaucine, tetrahydropalmatine, tetrahydrocoptisine, canadine, and corydaline) were detected in the [M+H]^+^ form, two components (columbamine and dehydrocorydaline) were detected in the [M]^+^ form, and the remaining four components (coptisine Cl, berberrubine Cl, palmatine Cl and berberine Cl) were detected in the [M–Cl]^+^ form (Table S3). The coefficient of determination value in the calibration curve of each compound tested at different concentrations was ≥0.9927, and the limits of detection and quantitation were 0.01–0.19 and 0.02–0.58 mg/L, calculated as signal-to-noise ratios of 3 and 10, respectively (Table S4). Using the established UPLC-MS/MS MRM analytical method, 13 compounds were detected at 0.002–18.40 mg/g in CTE, and dehydrocorydaline was the most abundant at 18.40 mg/g (Table 1).

### 2.2. Identification and Functional Enrichment Analysis of Differentially Expressed Genes (DEGs) in TI-Stimulated HaCaT Cells

**Figure 2:**
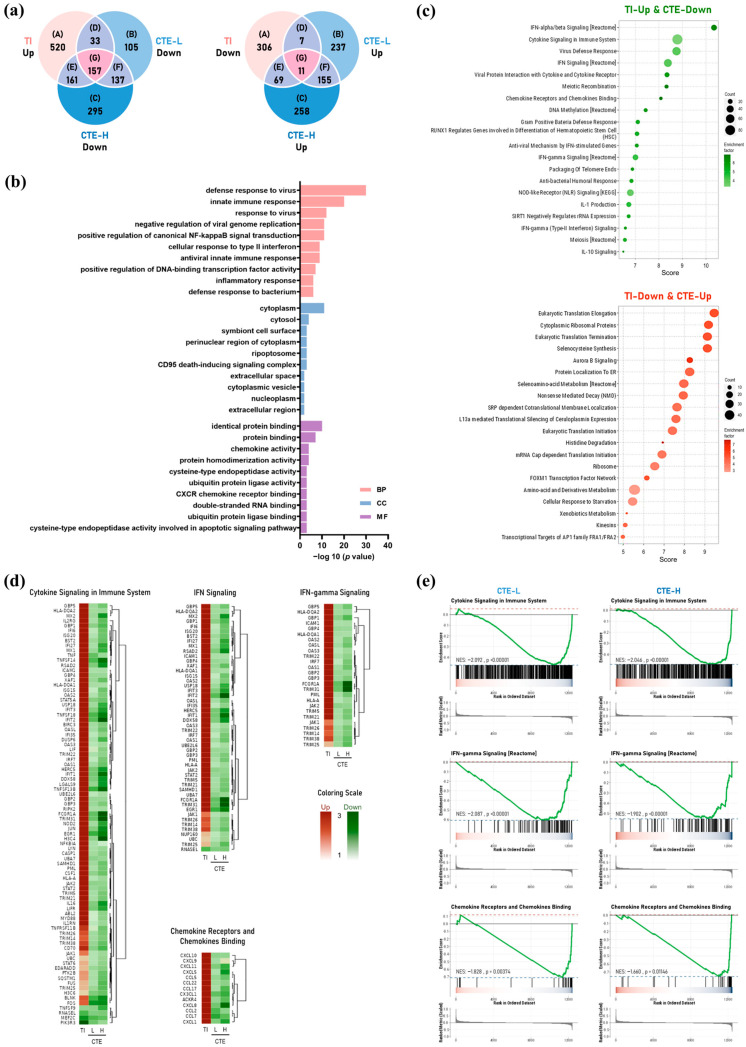
Identification and functional enrichment analysis of DEGs between TI and CTE in HaCaT cells. (**a**) Venn diagrams showing the common and specific DEGs among TI, CTE-L, and CTE-H. (**b**) GO terms implicated in downregulated DEGs that overlap between CTE-L and CTE-H compared to TI. (**c**) The top 20 enrichment pathways affected by CTE. The count of genes is shown by the size of the dots, and the enrichment factor is represented by the color of the dots. Compared to TI, the green and red colors indicate downregulated and upregulated DEGs, respectively. (**d**) Heatmap of DEGs associated with enriched pathways between TI and CTE. The green and red colors indicate downregulated and upregulated DEGs, respectively, compared to TI. (**e**) GSEA of the enriched pathways between TI and CTE. BP, biological process; CC, cell component; CTE, Corydalis Tuber 70% ethanol extract; CTE-L, CTE 25 μg/mL; CTE-H, CTE 100 μg/mL; DEGs, differentially expressed genes; GO, Gene Ontology; GSEA, gene set enrichment analysis; MF, molecular function; NES, normalized enrichment score; TI, TNF-α (10 ng/mL) and IFN-γ (10 ng/mL).

Results of cytotoxicity testing of CTE in HaCaT cells revealed an approximately 22% decrease in cell viability at a concentration of 200 μg/mL (Figure S2). All subsequent experiments with HaCaT cells were performed at concentrations of 12.5–100 μg/mL, which did not result in cytotoxicity. Transcriptomic analysis identified a total of 1264 DEGs in the TI group, including 871 upregulated and 393 downregulated genes based on the thresholds of |logFC| > 1.3 and *p* < 0.01 (Figure 2a). The list of DEGs, including upregulated and downregulated genes, was as previously published [16]. The overlapping genes between tumor necrosis factor (TNF)-α and interferon (IFN)-γ (TI), CTE 25 μg/mL (CTE-L), and CTE 100 μg/mL (CTE-H) are represented in a Venn diagram (Figure 2a), and the list of genes included in each region is presented in the Supplementary Materials-DEGs list. Compared with TI, 157 downregulated genes overlapping between CTE-L and CTE-H were shown to be enriched in “innate immune response”, “positive regulation of canonical NF-kappaB signal transduction”, and “inflammatory response” for biological process (BP) and “chemokine activity” and “CXCR chemokine receptor binding” for molecular function (MF) (Figure 2b). The top 20 enriched pathways were showed as dotplots (Figure 2c). Among these pathways, the expression patterns of DEGs belonging to the top-ranked “Cytokine Signaling in Immune System”, “IFN Signaling”, “IFN-gamma Signaling”, and “Chemokine Receptors and Chemokine Binding”; pathways were visualized as a heatmap of hierarchical clustering analysis (Figure 2d), and gene set enrichment analysis (GSEA) indicated that DEGs were mainly involved in these pathways (Figure 2e).

### 2.3. Effects of CTE and Its Compounds on Chemokine Production and Phosphorylated-Janus Kinase (JAK), Signal Transducers and Activators of Transcription (STAT), and Nuclear Factor Kappa-Light-Chain-Enhancer of Activated B Cells (NF-κB) Expression in TI-Stimulated HaCaT Cells

**Figure 3:**
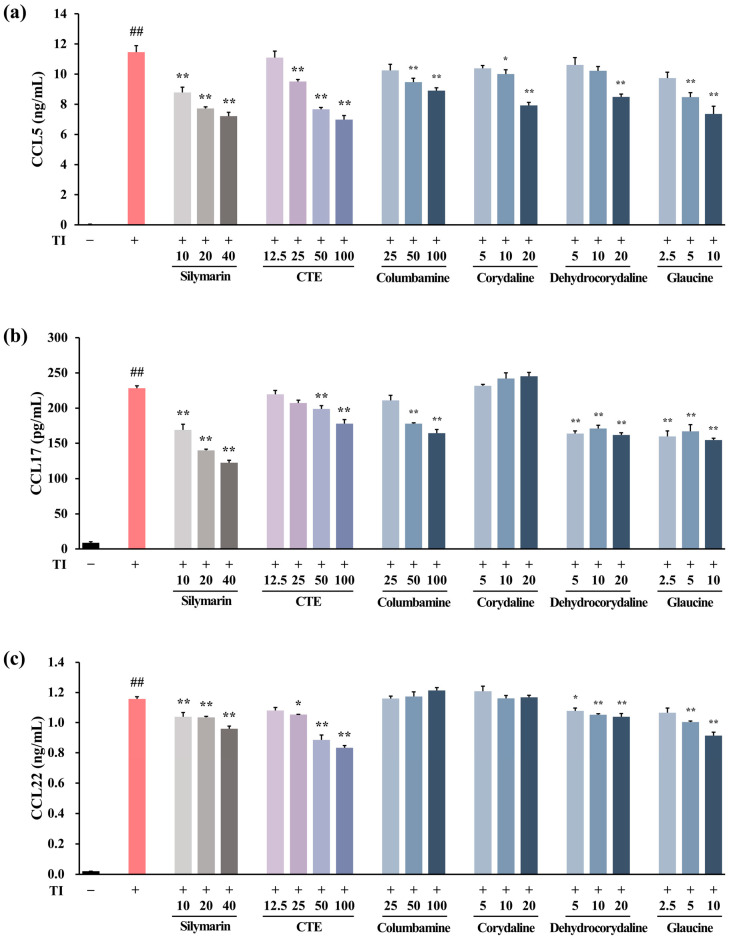
Effect of CTE and its compounds on chemokine production in TI-stimulated HaCaT cells. The cells were treated with CTE or its compounds and stimulated with TI for 24 h. The levels of CCL5 (**a**), CCL17 (**b**), and CCL22 (**c**) in the supernatant were measured. Silymarin was used as a positive control. Data are expressed as means ± SEMs (*n* = 3). ^##^ *p* < 0.01 vs. normal control; * *p* < 0.05 and ** *p* < 0.01 vs. TI-stimulated cells. CTE, Corydalis Tuber 70% ethanol extract; TI, TNF-α (10 ng/mL) and IFN-γ (10 ng/mL).

**Figure 4:**
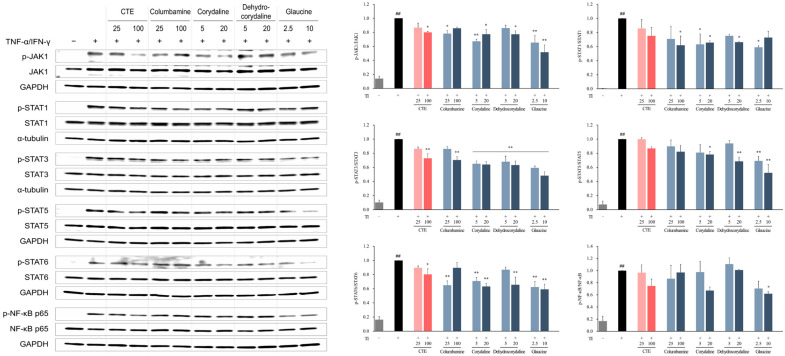
Effect of CTE and its compounds on the JAK-STAT and NF-κB pathways in TI-stimulated HaCaT cells. The cells were treated with CTE or its compounds and stimulated with TI for 30 min. Expression of total and phosphorylated (p-) JAK1, STAT1, STAT3, STAT5, STAT6, and NF-κB p65 in whole cell lysates was determined by Western blotting. Data are presented as means ± SEMs as the relative expression ratio of the phosphorylated form to the total form. ^##^ *p* < 0.01 versus normal control; * *p* < 0.05 and ** *p* < 0.01 versus TI-stimulated cells. CTE, Corydalis Tuber 70% ethanol extract; TI, TNF-α (10 ng/mL) and IFN-γ (10 ng/mL).

Based on the transcriptome analysis results showing that CTE was involved in “immune and inflammatory responses” and “chemokine activity”, screening was conducted to observe whether chemokine production was reduced in TI-stimulated HaCaT cells. TI treatment significantly increased the production of C-C motif chemokine ligand (CCL5), CCL17, and CCL22 in HaCaT cells when compared to the normal control (NC) (*p* < 0.01, Figure 3a–c). Silymarin, a positive control, significantly inhibited CCL5, CCL17, and CCL22 levels in a dose-dependent manner when compared with TI-treated cells (*p* < 0.01). CTE (12.5–100 μg/mL) suppressed TI-induced CCL5, CCL17, and CCL22 production (*p* < 0.01) in a dose-dependent manner. The 13 compounds included in CTE were treated with HaCaT cells at non-toxic concentrations (Figure S2), and four components were selected as active ingredients (Figure S3). Among the compounds included in CTE, columbamine significantly inhibited the levels of CCL5 and CCL17 when compared to the TI (*p* < 0.01). Corydaline reduced the TI-induced CCL5 release (*p* < 0.01) but had no effect on CCL17 and CCL22 secretion. Treatment with dehydrocorydaline and glaucine decreased the TI-induced CCL5, CCL17, and CCL22 production (*p* < 0.01). To determine the mechanism of CTE on the chemokine release inhibition, we performed Western blot analysis to examine the expression of JAK-STAT and NF-κB associated with DEGs, which were enriched in the top-ranked pathways in transcriptome analysis. Compared to TI, treatment with CTE and its four active components—columbamine, corydaline, dehydrocorydaline, and glaucine—significantly suppressed the phosphorylation of JAK1, STAT3, and STAT6 (*p* < 0.05 or *p* < 0.01, Figure 4). The phosphorylation of STAT1 was inhibited by four active compounds (*p* < 0.05) and reduced by CTE, but this effect was not statistically significant. TI-induced STAT5 and NF-κB activation was decreased by CTE, although the difference was not statistically significant. Corydaline and dehydrocorydaline downregulated the STAT5 phosphorylation (*p* < 0.05 or *p* < 0.01), while glaucine reduced the NF-κB activation (*p* < 0.05).

### 2.4. Effects of Topical Application of CTE on HDM-Induced AD Symptoms in NC/Nga Mice

**Figure 5:**
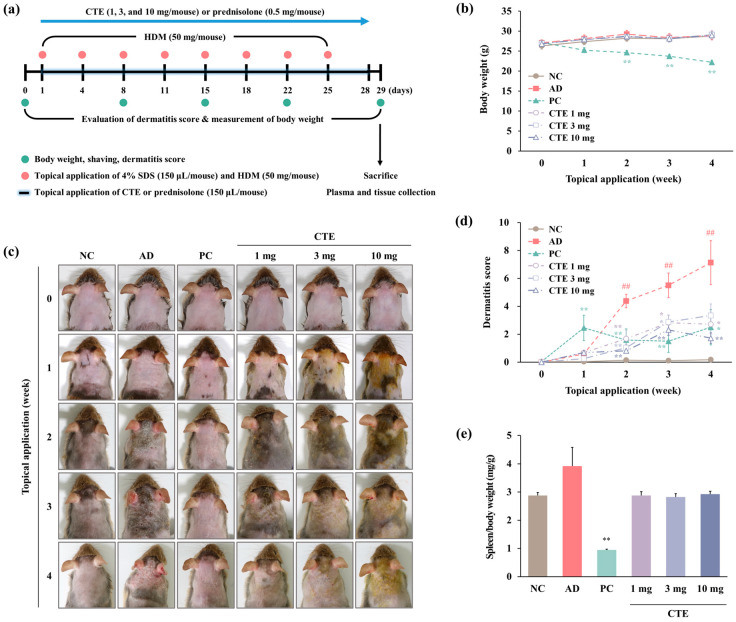
Effect of CTE on macroscopic changes in HDM-induced AD mice. (**a**) Schematic diagram of the experimental schedule. AD was induced by treating the dorsal skin and ears of mice with 4% SDS (150 μL/mouse) and HDM (50 mg/mouse) twice a week for 4 weeks. CTE (1, 3, and 10 mg/mouse) and prednisolone (0.5 mg/mouse) were applied topically every day for 4 weeks. (**b**) Changes in body weight from each group. (**c**) Images of the skin lesions on each mouse. (**d**) Dermatitis scores were assessed once weekly for 4 weeks. Dermatitis scores represent sums of individual scores graded as follows: 0 (none), 1 (mild), 2 (moderate), and 3 (severe) for each of the four symptoms: erythema/hemorrhage, scarring/dryness, edema, and excoriation/erosion. (**e**) Spleen weight relative to body weight from each group. Data are expressed as means ± SEMs (*n* = 6–8). ^##^ *p* < 0.01 vs. NC; * *p* < 0.05 and ** *p* < 0.01 vs. AD. AD, atopic dermatitis; CTE, Corydalis Tuber 70% ethanol extract; HDM, house dust mite (Biostir-AD^®^ ointment; Biostir Inc., Osaka, Japan); NC, normal control; PC, positive control.

In NC/Nga mice induced with dermatitis for 4 weeks (Figure 5a), there was no change in body weight among the groups, except for the prednisolone group, which displayed a significant decrease when compared with the NC group from the second week of application (Figure 5b). In the HDM-induced AD group, dermatitis symptoms, such as erythema/hemorrhage, scarring/dryness, edema, and excoriation/erosion, worsened from the 2nd week, and the dermatitis score significantly increased when compared to that in NC group (*p* < 0.01, Figure 5c,d). These symptoms dramatically reduced upon topical treatment with CTE (1, 3, and 10 mg/mouse) and prednisolone (0.5 mg/mouse) when compared to those in the AD group (*p* < 0.05 or *p* < 0.01). The spleen weight relative to body weight increased in the AD group when compared to the NC group but decreased in the CTE-treated group to a level similar to that of the NC group (Figure 5e). In contrast, the spleen weight in the prednisolone group was significantly lower than that in the NC group (*p* < 0.01).

### 2.5. Effects of Topical Application of CTE on the Plasma Levels of Chemokines, Histamine, IgE, and Stress Hormones in HDM-Treated NC/Nga Mice

**Figure 6:**
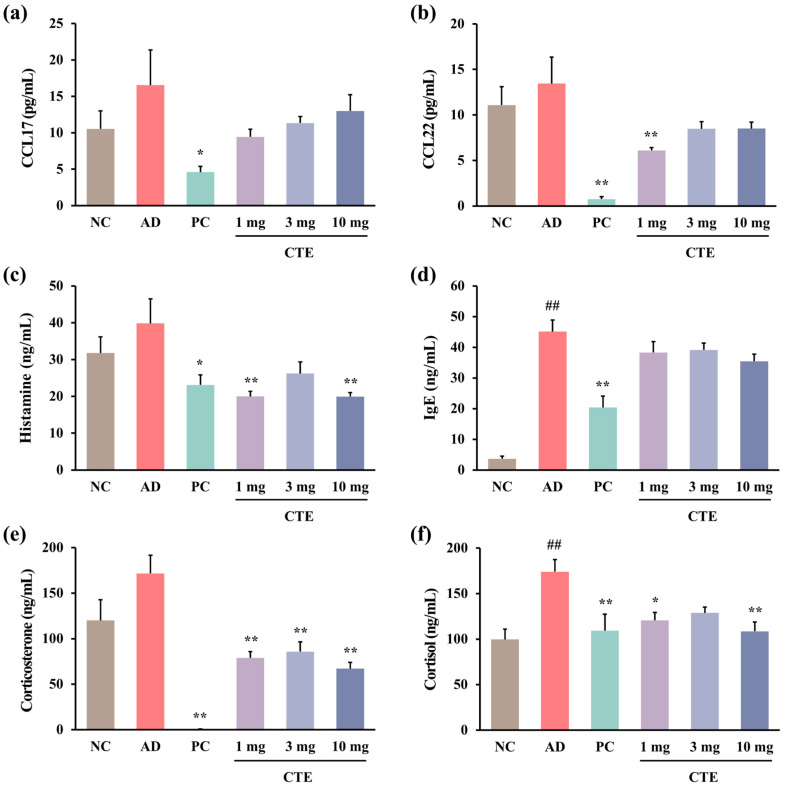
Effect of CTE on the plasma levels of biomarkers in HDM-induced AD mice. The levels of CCL17 (**a**), CCL22 (**b**), histamine (**c**), IgE (**d**), corticosterone (**e**), and cortisol (**f**) in plasma were measured using ELISA. Data are expressed as means ± SEMs (*n* = 6–8). ^##^ *p* < 0.01 vs. NC; * *p* < 0.05 and ** *p* < 0.01 vs. AD. AD, atopic dermatitis; CTE, Corydalis Tuber 70% ethanol extract; HDM, house dust mite (Biostir-AD^®^ ointment; Biostir Inc., Osaka, Japan); NC, normal control; PC, positive control.

CCL17, CCL22, and histamine levels in the plasma increased with HDM treatment, but the difference was not statistically significant (Figure 6a–c). In contrast to the AD group, topical CTE application restored these levels back to normal. Plasma IgE levels in the AD group were considerably higher than those in the NC group (*p* < 0.01), and they tended to decrease with topical CTE treatment; however, the difference was not significant (Figure 6d). Stress-related plasma corticosterone and cortisol levels were higher in the AD group than those in the NC group, whereas these levels were significantly reduced in the CTE treatment group (Figure 6e,f). All these factors were significantly lower in the prednisolone group than in the AD group (*p* < 0.05 or *p* < 0.01).

### 2.6. Effects of Topical Application of CTE on the Histopathological Alteration in HDM-Treated NC/Nga Mice

**Figure 7:**
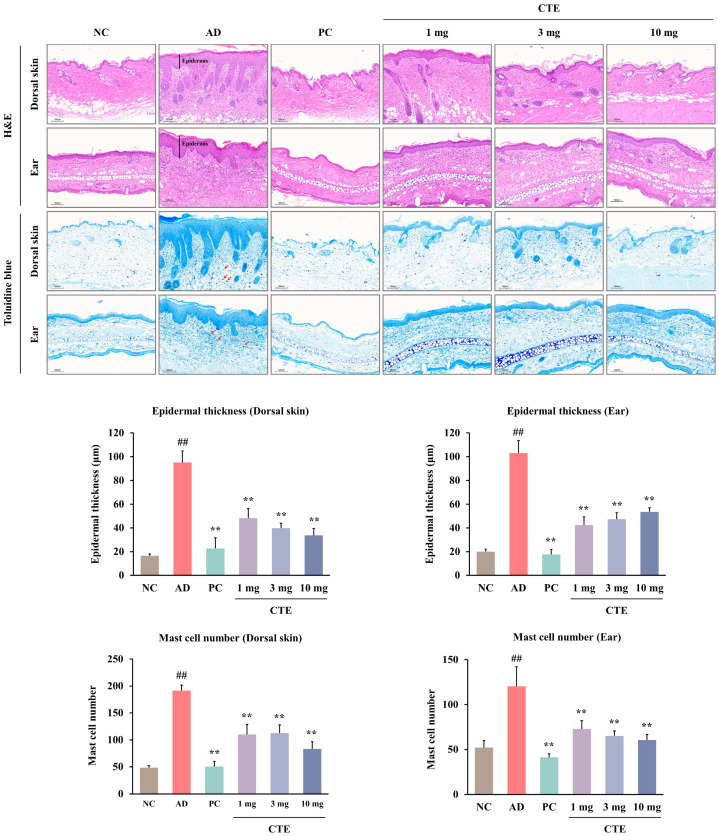
Effect of CTE on the histopathological alterations and mast cell infiltration of dorsal skin and ear lesions in HDM-induced AD mice. Histopathological features of lesions were identified by H&E staining (×10). On the H&E staining slide, the epidermal thickness of the dorsal skin and ear (the black line) was measured in three randomly selected regions. Mast cells were stained with toluidine blue (×10). The number of purple-stained mast cells (the red arrow) on the toluidine blue-stained dorsal skin and ear was counted in two randomly selected regions. Data are presented as mean ± SEM (*n* = 6–8). ^##^ *p* < 0.01 vs. NC; ** *p* < 0.01 vs. AD. Scale bar = 100 μm. AD, atopic dermatitis; CTE, Corydalis Tuber 70% ethanol extract; H&E, hematoxylin and eosin; HDM, house dust mite (Biostir-AD^®^ ointment; Biostir Inc., Osaka, Japan); NC, normal control; PC, positive control.

Epidermal hyperplasia was observed in the dorsal skin and ear tissues of the AD group (Figure 7). Topical application of CTE (1, 3, and 10 mg) and prednisolone improved HDM-induced histopathological alterations. However, the epidermal thickness in the prednisolone group was abnormally thinner than that in the NC group. Toluidine blue (TB) staining confirmed the infiltration of mast cells into the dorsal skin and ear tissue lesions. The number of mast cells in the lesions of the AD group was significantly higher than that in the NC group (Figure 7). Mast cell infiltration into the lesions significantly decreased following treatment with CTE (1, 3, and 10 mg) and prednisolone.

### 2.7. Effects of Topical Application of CTE on the Expression of TSLP, CD4^+^ T Cells, Interleukin (IL)-4, and ICAM-1 in HDM-Treated NC/Nga Mice

**Figure 8:**
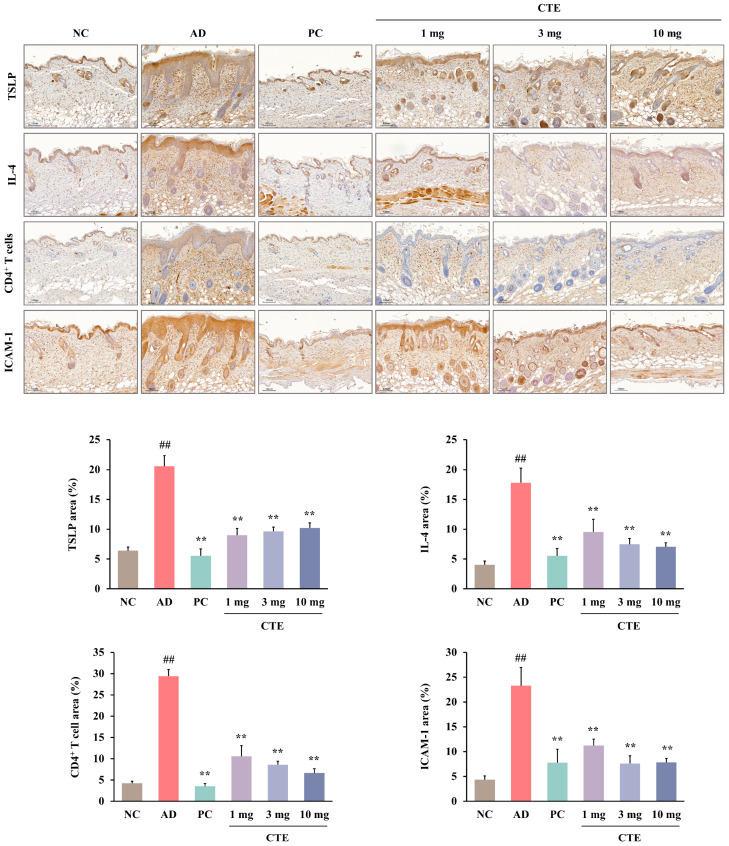
Immunohistochemical analysis of TSLP, CD4^+^ T cells, IL-4, and ICAM-1 in the dorsal skin of HDM-induced AD mice. A quantitative morphometric analysis was performed on the immunostained area in dark brown compared to the total area. Data are presented as means ± SEMs (*n* = 6–8). ^##^ *p* < 0.01 vs. NC; ** *p* < 0.01 vs. AD. Scale bar = 100 μm. AD, atopic dermatitis; CTE, Corydalis Tuber 70% ethanol extract; HDM, house dust mite (Biostir-AD^®^ ointment; Biostir Inc., Osaka, Japan); NC, normal control; PC, positive control.

Immunohistochemical (IHC) analysis revealed that the expression of TSLP, CD4^+^ T cells, IL-4, and ICAM-1 in skin lesions was significantly higher in the AD group than in the NC group (*p* < 0.01; Figure 8). CTE (1, 3, and 10 mg) and prednisolone significantly decreased the expression of TSLP, CD4^+^ T cells, IL-4, and ICAM-1 when compared to that in the AD group (*p* < 0.01). In particular, the effect of CTE 10 mg was similar to that of prednisolone.

## 3. Discussion

To the best of our knowledge, this is the first study to demonstrate the pharmacological effects of CTE on AD using in vitro and in vivo models. We observed that CTE reduced chemokine release by suppression of the JAK-STAT and NF-κB pathways in TI-stimulated HaCaT cells. Furthermore, our results showed that CTE improved AD symptoms both physiologically and histopathologically in an HDM-induced AD mouse model.

The pharmacological efficacy of herbal medicine can be attributed to the synergistic actions of various ingredients. Therefore, compound analysis is required to clarify the pharmacological basis of herbal medicines and improve quality control [17]. In the present study, we used UPLC-MS/MS to identify and quantify 13 compounds in CTE, including tetrahydrocolumbamine, protopine, columbamine, glaucine, coptisine, tetrahydropalmatine, tetrahydrocoptisine, berberrubine, canadine, corydaline, palmatine, berberine, and dehydrocorydaline. Coptisine [18] and berberine [19] can reduce psoriasis and dermatitis in vitro and in vivo, respectively. Dehydrocorydaline [20] and protopine [21,22] exhibit anti-type I-IV allergic reactions and anti-inflammatory properties, respectively. Considering the pharmacological activity of these components, CTE may be beneficial as an AD-related therapeutic target.

Our functional enrichment analysis of DEGs in TI-stimulated HaCaT cells revealed that DEGs downregulated by CTE when compared to TI are enriched in pathways such as “Cytokine Signaling in Immune System”, “IFN Signaling”, “IFN-gamma Signaling”, and “Chemokine Receptors and Chemokine Binding”. These pathways are involved in the development of AD. Keratinocytes secrete chemokines in response to mechanical damage to recruit T cells into the skin, and TSLP-activated dendritic cells differentiate into T cells. Activated T cells produce cytokines, such as IL-4, IL-5, IL-13, and IFN-γ, and the atopy-related chemokines CCL17 and CCL22. Cytokines maintain and promote chemokine production in lesions; in particular, IL-4 mediates IgE production in B cells [3,23]. In the present study, CTE treatment inhibited CCL5, CCL17, and CCL22 production in TI-stimulated HaCaT cells and reduced the plasma levels of CCL17 and CCL22 in HDM-induced AD mice. Additionally, the effects of CTE on the on JAK-STAT and NF-κB pathways were investigated based on the results of transcriptome analysis to identify its mechanism of action. The JAK-STAT and NF-κB pathways play a crucial role in immune function and mediate the signaling of important cytokines in the pathogenesis of AD [24,25,26]. It was determined that CTE inhibited the phosphorylation of JAK1, STAT3, and STAT6 in TI-stimulated HaCaT cells. The activated JAK phosphorylates the residues on cytokine receptors to recruit STAT proteins [24]. JAK1, which is involved in the development and regulation of the immune system [25], can phosphorylate all STAT families, which consist of seven proteins (STAT1, STAT2, STAT3, STAT4, STAT5A/B, and STAT6) [24]. Phosphorylated STAT is dimerized and translocated to the nucleus to induce the expression of cytokine-related genes [25]. Among the STAT family, STAT3 plays a central role in signal transduction from the plasma membrane to the nucleus and is mainly involved in the regulation of the immune system, cell growth, differentiation, development, and apoptosis [24,27]. STAT6, which is crucial for the biological activities of IL-4 and IL-13, is associated with B cell proliferation and maturation, IgE expression, and mast cell activation [25,27]. Considering the close association between JAK1-STAT3/STAT6 and the pathogenesis of AD, CTE may be a promising therapeutic candidate for AD. Additionally, it seems that CTE suppresses chemokines production by downregulating the JAK1-STAT3/STAT6 and NF-κB pathways. Coptisine [18], berberine [19], and protopine [21,22], previously reported to reduce dermatitis, psoriasis, allergic reactions, or inflammation, did not inhibit chemokine production in our in vitro study. This outcome could be attributed to the low, non-cytotoxic concentrations used in the experiments, necessitated by the strong cytotoxicity of these compounds in HaCaT cells. The concentrations of columbamine (0.41 μg/mL), corydaline (0.57 μg/mL), dehydrocorydaline (1.84 μg/mL), and glaucine (0.51 μg/mL) at 100 μg/mL, the maximum treatment concentration of CTE that suppresses chemokines in HaCaT cells, were below the effective concentrations for each individual component. Consequently, CTE appears to exert a synergistic effect through the combination of multiple compounds, including dehydrocorydaline, which is the most abundant component in CTE.

Based on these results, we investigated whether CTE affects AD-like lesions in HDM-treated NC/Nga mice. In the skin of CTE-treated groups, the expression levels of TSLP, CD4^+^ T cells, IL-4, and ICAM-1 were lower than those in the untreated AD group. Therefore, we suggest that CTE improves AD symptoms by complexly regulating multiple factors related to AD pathology, including chemokine secretion from keratinocytes, TSLP, IL-4, and ICAM-1 expression in lesions, and CD4^+^ T cell priming.

The hyperactivation of mast cells by IgE may induce allergic inflammation, resulting in the prolonged and chronic continuation of the inflammatory responses. In particular, mast cells secrete histamine, which causes itching and worsens dermatitis [3]. Our data revealed that CTE reduced plasma histamine levels and inhibited mast cell infiltration into lesions in HDM-induced AD mice.

Skin diseases influence behavior and emotion regulation in addition to physical conditions and pathological changes. Patients with AD exhibit lower vitality, social function, and mental health scores than the general population or patients with other diseases, such as diabetes or high blood pressure [28]. Our in vivo results revealed that the AD group experienced more stress, as shown by higher plasma corticosterone and cortisol concentrations, when compared to the NC group, which was reduced by CTE treatment. In previous studies, CTE has exhibited an antidepressant effect along with reducing stress hormone levels [10,11]; therefore, it may improve the vicious cycle of AD and stress.

Meanwhile, prednisolone decreased the levels of histamine, IgE, CCL17, and CCL22 in plasma, but had adverse effects, such as lowering body weight, skin atrophy, and smaller spleens, compared to the NC. Consequently, CTE, which does not cause any side effects, is considered to be safer than prednisolone. Our findings suggest that CTE may be a safe and useful candidate to replace or complement corticosteroids for the treatment of various inflammatory diseases, including AD. Although long-term efficacy and safety studies are required, topical application of CTE is predicted to be safe and effective for at least 4 weeks in future clinical trials or treatment options.

## 4. Materials and Methods

### 4.1. Plant Materials and Preparation of CTE

Dried Corydalis Tuber, sourced from China, was obtained from Kwangmyeongdang (Ulsan, Republic of Korea), a specialized herbal medicine provider, in April 2022. Morphological identification of the plant material was conducted by Dr. Goya Choi of the Korea Institute of Oriental Medicine (KIOM). First, 500 g of dried Corydalis Tuber was immersed in 1.0 L of 70% ethanol (EtOH). Next, ultrasonic extraction, comprising three cycles of 1 h each, was performed at room temperature (23 ± 2 °C). Subsequently, the extract was filtered through a standard 270-mesh sieve, followed by removal of the organic solvent using a rotary evaporator (EV-1020, SciLab Korea Co., Ltd., Wonju, Republic of Korea). The concentrated extract was lyophilized using a freeze dryer (PVTFD-100, ilShinBioBase Co., Ltd., Yangju, Republic of Korea). The sequential procedure yielded 29.3 g of powdered extract (manufacturer’s serial number: K1562210414; specimen number: EBM156; yield: 5.9%).

### 4.2. Chemicals and Reagents

The compounds (Figure 1a) selected for simultaneous determination in CTE using UPLC-MS/MS in CTE were purchased from reputable natural product manufacturers: tetrahydrocolumbamine (CAS No. 483-34-1, catalog No. TT033202, 98.0%) and protopine (CAS No. 130-86-9, catalog No. TB0803-0025, 98.4%) were obtained from Wuhan ChemNorm Biotech Co., Ltd. (Wuhan, China); columbamine (CAS No. 3621-36-1, catalog No. DR11751, 99.3%), glaucine (CAS No. 475-81-0, catalog No. DR11753, 99.0%), coptisine chloride (Cl) (CAS No. 6020-18-4, catalog No. DR10790, 98.3%), tetrahydrocoptisine (CAS No. 4312-32-7, catalog No. DR11749, 99.6%), berberrubine Cl (CAS No. 15401-69-1, catalog No. DR11748, 98.3%), canadine (CAS No. 522-97-4, catalog No. DR11750, 99.2%), corydaline (CAS No. 518-69-4, catalog No. DR10984, 99.8%), berberine Cl (CAS No. 633-65-8, catalog No. DR10793, 98.9%), and dehydrocorydaline (CAS No. 30045-16-0, catalog No. DR10986, 98.6%) were obtained from Shanghai Sunny Biotech Co., Ltd. (Shanghai, China); palmatine Cl (CAS No. 10605-02-4, catalog No. P2138, 99.3%) was obtained from Tokyo Chemical Industry Co., Ltd. (Tokyo, Japan); tetrahydropalmatine (CAS No. 483-14-7, catalog No. CFN99195, 99.3%) was purchased from ChemFaces (Wuhan, China). Detailed information regarding the chemicals and reagents used in both in vitro and in vivo experimental procedures is available in the Supplementary Materials (Table S1).

### 4.3. UPLC-MS/MS Simultaneous Analysis of Compounds in CTE

The 13 compounds in CTE were determined simultaneously using a UPLC-MS/MS system comprising an Acquity UPLC system and a Xevo TQ-XS mass spectrometry (MS) system (Waters, Milford, MA, USA). Detailed conditions for the UPLC-MS/MS analysis of the compounds and parameters are presented in Supplementary Materials (Tables S2 and S3 and Figure S1).

### 4.4. Cell Culture and Viability Assay

HaCaT cell line was acquired from CLS Cell Lines Service GmbH. HaCaT cells were maintained in Dulbecco’s modified Eagle’s medium, supplemented with 10% fetal bovine serum and antibiotics (100 U/mL penicillin and 100 μg/mL streptomycin). Cells were maintained at 37 °C in a humidified atmosphere with 5% CO_2_ and 95% air. To assess the cytotoxic effects of CTE, the cells were exposed to various concentrations of CTE for 24 h, and cell viability was quantified using a Cell Counting Kit-8, following the manufacturer’s instructions.

### 4.5. Transcriptome and DEG Analysis in HaCaT Cells

For transcriptome analysis, the cells were treated with different concentrations of CTE in the presence of TNF-α and IFN-γ (each 10 ng/mL) for 24 h. Total RNA was extracted from cells at DNA Link Inc. (Seoul, Republic of Korea), and RNA-sequencing was conducted at KaiPharm Co., Ltd. (Seoul, Republic of Korea), as described previously [16]. DEG analysis was conducted using the DESeq2 software (Version 1.38.2). Genes with a |logFC| > 1.3 and *p*-value < 0.01 were considered as DEGs. To elucidate the functional implications of DEGs, overlapping genes between TI and the CTE-L and CTE-H groups were mapped to Gene Ontology (GO) terms using the DAVID functional annotation tool: BP, cell component (CC), and MF [29]. The enriched genes associated with each GO term were then quantified. To analyze the possible biological functions and enriched pathways of DEGs, public databases, including the Kyoto Encyclopedia of Genes and Genomes, GO biological process, Molecular Signature Database, Chemical Genetic Perturbation, Oncogenic Signature, EMTome, and Secretome, were utilized. Subsequently, the datasets were subjected to over-representation analysis and GSEA. The significantly enriched gene sets were identified based normalized enrichment scores and combined *p*-values using the fgsea package in R (Version 4.2.1, R Software for Statistical Computing, Vienna, Austria).

### 4.6. Measurement of Chemokine Levels in HaCaT Cells

The influence of CTE on chemokine release was determined using culture supernatants, as explained in the section on “Transcriptome and DEGs analysis in HaCaT cells”. The levels of CCL5 (regulated on activation, normal T cell expressed, and secreted; RANTES), CCL17 (thymus- and activation-regulated chemokines; TARC), and CCL22 (macrophage-derived chemokines) in the culture supernatants were measured via enzyme-linked immunosorbent assay (ELISA). Silymarin was used as a positive control, as described previously [30].

### 4.7. Western Blot

The alterations in the JAK 1, STATS (STAT1, STAT3, STAT5, and STAT6), and NF-κB signaling pathways were verified using Western blotting to examine the anti-AD mechanism of action of CTE in HaCaT cells. The whole procedure was carried out as described previously [16]. Information about the antibodies is provided in the Supplementary Materials (Supplementary Tables S1 and S2). We calculated *p*-values using one-way ANOVA, then performed Fisher’s Least Significant Difference test using GraphPad Prism (Version 9.5.1, GraphPad Software, LLC, Boston, MA, USA). A *p*-value < 0.05 indicated significance.

### 4.8. Animals and Treatment

Male, 9-week-old specific pathogen-free NC/Nga mice were obtained from Central Laboratory Animal Inc. (Seoul, Republic of Korea). Animals were housed in a controlled environment with a temperature range of 20–24 °C, relative humidity maintained at 40–60%, an average ventilation frequency of 20 times per hour, and a 12-h light/dark cycle with an illumination intensity of 250–350 lx. They were acclimatized for 1 week before the initiation of the experiment, with ad libitum access to water and feed provided throughout the study. This study followed the NIH Guidelines for the Care and Use of Laboratory Animals [31] and was approved by the Institutional Animal Care and Use Committee of the KIOM (approval number: #22-076). The animals were divided into six groups, each consisting of 6–8 individuals: NC, AD, positive control (PC) treated with prednisolone (0.5 mg/mouse), and experimental groups treated with CTE (1, 3, and 10 mg/mouse). The CTE dose was set to 2% (3 mg/mouse) [32], which is the content of topical ointments mainly used in clinical practice, and additionally, 1/3 (1 mg/mouse) and about 3 times (10 mg/mouse) were selected. The day before the experiment, the upper back was shaved. To induce AD-like skin lesions, HDM extract (Biostir-AD^®^ ointment; Biostir Inc., Osaka, Japan) [33] was topically applied twice weekly to the dorsal skin and both surfaces of each ear for 4 weeks, excluding the NC group, as previously described [34]. The NC and AD groups were topically treated with 70% EtOH, and the PC and CTE groups were topically treated with solutions containing prednisolone and CTE dissolved in 70% EtOH, respectively, once daily for four weeks. Body weight was measured weekly. The mice received an intraperitoneal injection of pentobarbital (Entobar) to induce euthanasia 18 h following the last treatment, and blood and tissue samples were collected. The dorsal skin and ears were fixed in 10% neutral buffered formalin for histopathological and IHC analysis.

### 4.9. Evaluation of Dermatitis Severity

The severity of dermatitis was determined macroscopically once weekly in a blinded manner, as previously described [34]. Erythema/hemorrhage, scarring/dryness, edema, and excoriation/erosion were scored from 0 (none) to 3 (severe), and the total score was calculated.

### 4.10. Measurement of Plasma Chemokines, Histamine, IgE, and Stress Hormone Levels

Plasma CCL17, CCL22, histamine, total IgE, corticosterone, and cortisol levels were measured using ELISA kits, following the manufacturer’s instructions.

### 4.11. Histopathological and IHC Analysis

To investigate histopathological characteristics, paraffin-embedded tissues of the dorsal skin and ear were sliced and subjected to hematoxylin and eosin (H&E) staining. The epidermal thickness in both the dorsal skin and ear was assessed by measuring three randomly selected regions on H&E-stained slides using Motic VM 3.0-Motic Digital Slide Assistant (Motic China Group Co., Ltd., Xiamen, China) with Motic EasyScan (Motic, Hong Kong). Furthermore, the dorsal skin and ear sections were stained using TB to identify the infiltrated mast cell in the stratum spinosum and basal layers. On the TB-stained slides, the number of purple-stained mast cells was counted in two randomly selected regions. The expression of TSLP, CD4^+^ T cells, IL-4, and ICAM-1 in dorsal skin was confirmed using IHC. All stained slides were visualized using Motic EasyScan, and quantitative analysis of the dark brown immunostained area over the entire region was performed using MetaMorph Offline (Molecular Devices, Inc., San Jose, CA, USA).

### 4.12. Statistical Analysis

Data were analyzed using one-way analysis of variance, followed by a post-hoc Bonferroni test with the SYSTAT program (Version 13.1, SYSTAT Software, Inc., San Jose, CA, USA). The level of significance was set at *p* < 0.05.

## 5. Conclusions

Our study demonstrates that CTE treatment ameliorates skin inflammation in vitro and AD symptoms in vivo. First, CTE treatment inhibited CCL5, CCL17, and CCL22 levels by blocking the JAK-STAT and NF-κB pathways in TI-stimulated HaCaT cells. Second, topical application of CTE attenuated HDM-induced AD symptoms, including dermatitis score, dorsal skin and ear epidermal thickness; plasma histamine, CCL17, CCL22, corticosterone, and cortisol levels; lesional TSLP, CD4^+^ T cells, IL-4, ICAM-1 expression; and infiltration of inflammatory cells into the lesions. The effect of CTE in alleviating AD was likely due to the synergistic action of the CTE compounds. Taken together, our findings suggest that CTE can be a potential therapeutic agent for AD. Additionally, this study may serve as a basis for future studies on the applicability of CTE in other inflammatory skin diseases.

## Figures and Tables

**Table 1:** Quantification of compounds in CTE using the UPLC-MS/MS MRM analytical method.

Code No.	Name	Amount (mg/g)	RSD (%)
1	Tetrahydrocolumbamine	3.48 ± 0.16	4.51
2	Protopine	5.26 ± 0.11	2.07
3	Columbamine	4.05 ± 0.11	2.61
4	Glaucine	5.10 ± 0.06	1.12
5	Coptisine Cl	10.44 ± 0.13	1.26
6	Tetrahydropalmatine	5.51 ± 0.06	1.07
7	Tetrahydrocoptisine	1.42 ± 0.04	2.54
8	Berberrubine Cl	0.02 × 10^−1^ ± 0.01 × 10^−2^	2.88
9	Canadine	3.39 ± 0.03	0.94
10	Corydaline	5.71 ± 0.02	0.34
11	Palmatine Cl	6.86 ± 0.07	0.99
12	Berberine Cl	6.47 ± 0.20	3.17
13	Dehydrocorydaline	18.40 ± 0.17	0.91

Data are presented as means ± standard deviations (*n* = 3). CTE, Corydalis Tuber 70% ethanol extract; MRM, multiple reaction monitoring; RSD, relative standard deviation; UPLC-MS/MS, ultra performance liquid chromatography with tandem mass spectrometry.

## Data Availability

Data are contained within the article and Supplementary Materials.

## Supplementary Materials

The following supporting information can be downloaded at: https://www.mdpi.com/article/10.3390/ijms26031291/s1.
