# Supplementary material for: Corydalis Tuber Extract Alleviates Atopic Dermatitis: Transcriptomics-Based Mechanism Prediction and In Vitro/In Vivo Studies
Source: Int J Mol Sci. 2025 Feb 3;26(3):1291. doi: 10.3390/ijms26031291 (PMC11818904; doi:10.3390/ijms26031291)
Supplement: Supplementary file 1 [file ijms-26-01291-s001.zip › Supplementary Materials/Supplementary Materials.pdf]

Supplementary Materials

Table S1. Information on chemicals and reagents used for *in vitro* and *in vivo* experiments.

Category	Chemicals and reagents	Company
Cell line	HaCaT	CLS Cell Lines Service GmbH (Eppelheim, Baden-Württemberg, Germany)
Cell culture	DMEM<br>FBS<br>Penicillin and streptomycin	Thermo Fisher Scientific (Waltham, MA, USA)
Reagent	Acetonitrile (LC–MS grade)<br>Methanol (LC–MS grade)<br>Water (LC–MS grade)	Fisher Scientific Co., LLC (San Jose, CA, USA)
	Ammonium formate	Kanto Chemical Co., Inc. (Tokyo, Japan)
	Prednisolone<br>Silymarin	Sigma-Aldrich (St. Louis, MO, USA)
	Biostir-AD®	Biostir Inc. (Osaka, Japan)
	Entobar (pentobarbital sodium)	Hanlim Pharm. Co., Ltd. (Seoul, Republic of Korea)
	Formic acid	Fujifilm Wako Pure Chemical Co., Ltd. (Osaka, Japan)
	IFN-γ<br>TNF-α	R&D Systems Inc. (Minneapolis, MN, USA)
	10% Neutral-buffered formalin	BBC Biochemical (WA, USA)

Assay kit	CCK-8	Dojindo (Kumamoto, Japan)
	Corticosterone<br>Cortisol	MyBioSource, Inc. (San Diego, CA, USA)
	Histamine	Oxford Biomedical Research Inc. (MI, USA)
	IgE	Abcam (Cambridge, UK & Cambridge, Massachusetts, USA)
	MDC<br>RANTES<br>TARC<br>TNF-α	R&D Systems Inc. (Minneapolis, MN, USA)
Antibody	$CD4^+$ T cell<br>TSLP	Abcam (Cambridge, UK & Cambridge, Massachusetts, USA)
	ICAM-1<br>IL-4	Biorbyt Ltd. (Cowley Road, Cambridge, United Kingdom)

Table S2. UPLC-MS/MS MRM analysis conditions for simultaneous quantification of compounds in CTE.

UPLC conditions				MS conditions	
System	Acquity UPLC I-Class			System	Xevo TQ-XS
Column	Acquity UPLC BEH C18 column (2.1 mm × 100 mm, 1.7 μm)			Software	MassLynx v4.2
Column temp.	45℃			Ion source	ESI positive (+)
Sample temp.	5℃			Acquisition mode	MRM
Injection volume	2.0 μL			Capillary voltage	1.2 kV
Flow rate	0.3 mL/min			Cone gas flow	50 L/h
Mobile phase A	0.1% (v/v) aqueous formic acid with 5 mM ammonium formate			Desolvation gas flow	700 L/h
Mobile phase B	Acetonitrile			Desolvation temperature	500℃
	Time (min)	A (%)	B (%)	Source temperature	150℃
	Initial	80	20		
	0.1	80	20		
Gradient	14.0	5	95		
	15.0	0	100		
	15.1	80	20		
	18.0	80	20		

CTE, Corydalis Tuber 70% ethanol extract; ESI, electrospray ionization; MRM, multiple reaction monitoring; UPLC-MS/MS, ultra-performance liquid chromatography-tandem mass spectrometry.

Table S3. UPLC-MS/MS MRM conditions for simultaneous analysis of compounds in CTE.

Code No.	Name	Ion mode	Molecular weight	MRM transition		Cone voltage (V)	Collision energy (eV)	Retention time (min)
				Precursor ion	Product ion			
1	Tetrahydrocolumbamine	+	341.4	342.3	178.0	30	25	2.07
2	Protopine	+	353.4	354.3	188.0	30	30	2.39
3	Columbamine	+	338.4	338.3	322.0	30	30	2.54
4	Glaucine	+	355.4	356.3	293.9	30	20	2.64
5	Coptisine Cl	+	355.8	320.3	292.0	45	25	2.68
6	Tetrahydropalmatine	+	355.4	356.4	192.1	30	25	2.70
7	Tetrahydrocoptisine	+	323.3	324.3	176.0	30	30	2.85
8	Berberrubine Cl	+	357.8	322.2	307.1	30	30	2.90
9	Canadine	+	339.4	340.5	176.0	30	30	3.02
10	Corydaline	+	369.5	370.3	192.1	30	30	3.18
11	Palmatine Cl	+	387.9	352.3	336.0	40	30	3.25
12	Berberine Cl	+	371.8	336.3	320.0	35	30	3.34
13	Dehydrocorydaline	+	366.4	366.6	350.0	30	30	3.61

CTE, Corydalis Tuber 70% ethanol extract; MRM, multiple reaction monitoring; UPLC-MS/MS, ultra-performance liquid chromatography-tandem mass spectrometry.

Table S4. Parameters for simultaneous quantification of compounds in CTE using the UPLC-MS/MS MRM method.

Code No.	Name	Linear range (μg/L)	Regression equation $y = ax + b$	r^2	LOD (μg/L)	LOQ (μg/L)
1	Tetrahydrocolumbamine	5 – 500	$y = 127390x - 120495$	0.9981	0.01	0.03
2	Protopine	5 – 500	$y = 29438x - 58058.8$	0.9971	0.03	0.08
3	Columbamine	10 – 1,000	$y = 43294x - 295391$	0.9964	0.19	0.58
4	Glaucine	5 – 500	$y = 80863x - 198295$	0.9985	0.02	0.06
5	Coptisine Cl	50 – 5,000	$y = 6147x - 119463$	0.9959	0.05	0.15
6	Tetrahydropalmatine	5 – 500	$y = 159174x - 505675$	0.9969	0.01	0.03
7	Tetrahydrocoptisine	5 – 500	$y = 133471x - 303105$	0.9995	0.01	0.04
8	Berberrubine Cl	5 – 500	$y = 122839x - 491686$	0.9962	0.04	0.12
9	Canadine	10 – 1,000	$y = 72227x - 691221$	0.9927	0.03	0.08
10	Corydaline	10 – 1,000	$y = 63976x - 236447$	0.9989	0.18	0.54
11	Palmatine Cl	10 – 1,000	$y = 92806x - 450835$	0.9965	0.01	0.02
12	Berberine Cl	50 – 5,000	$y = 19982x - 682596$	0.9991	0.03	0.08
13	Dehydrocorydaline	10 – 1,000	$y = 39203x - 183104$	0.9987	0.06	0.18

CTE, Corydalis Tuber 70% ethanol extract; LOD, limit of detection; LOQ, limit of quantitation; MRM, multiple reaction monitoring; UPLC-MS/MS, ultra-performance liquid chromatography-tandem mass spectrometry; *x*, concentration (μg/L) of each reference compound; *y*, peak area of each reference compound.

Figure S1. Precursor ion (Q1, left) and product ion (Q3, right) chromatograms of each reference standard compound by UPLC-MS/MS MRM mode.

Tetrahydrocolumbamine

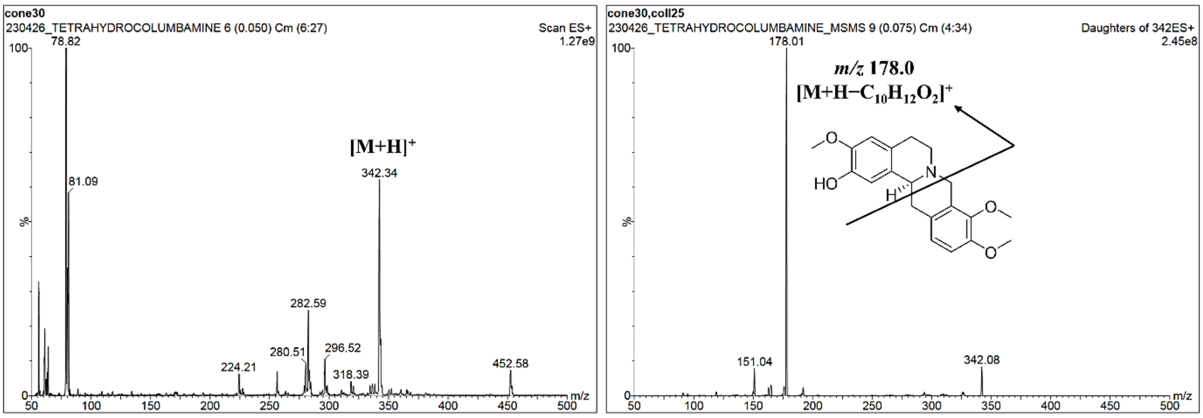

Protopine

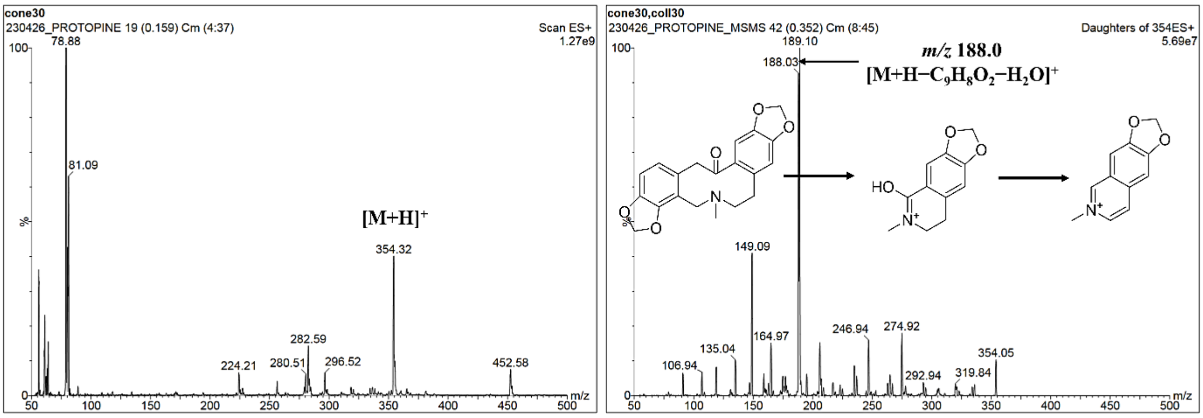

Columbamine

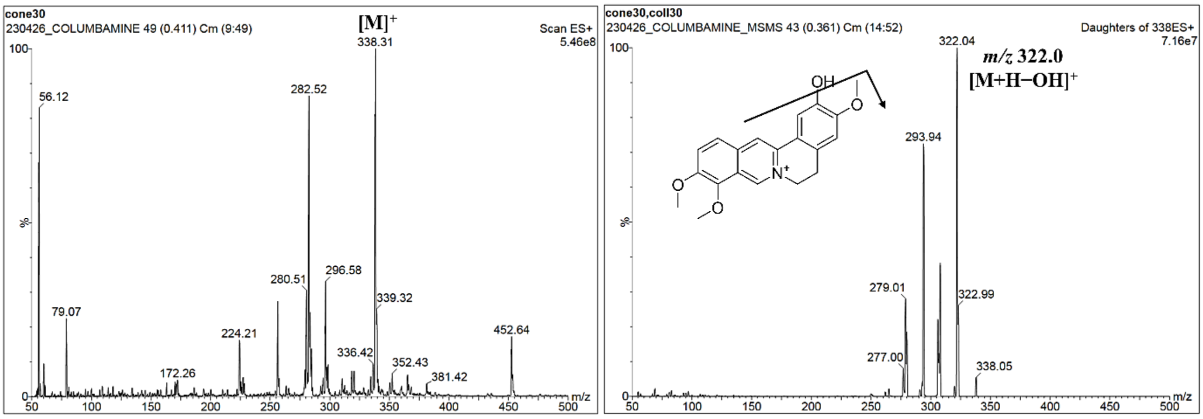

Glaucine

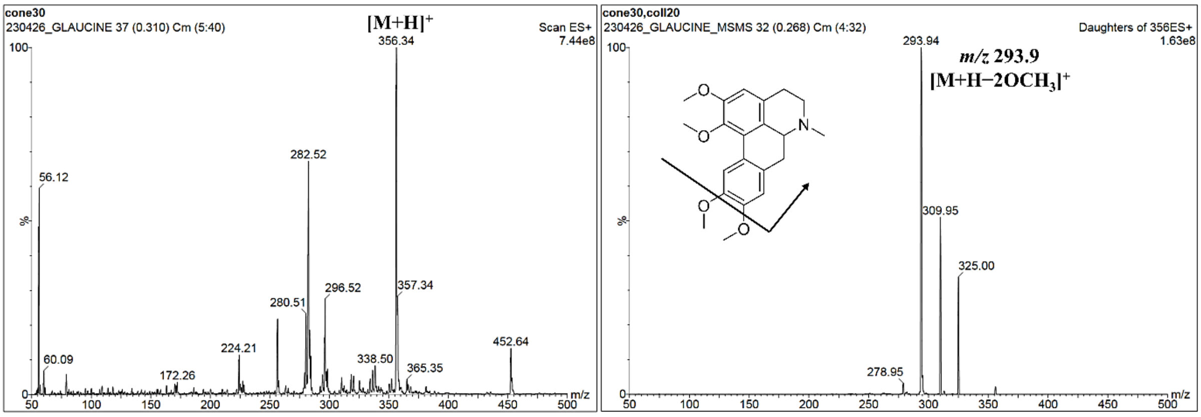

Coptisine Cl

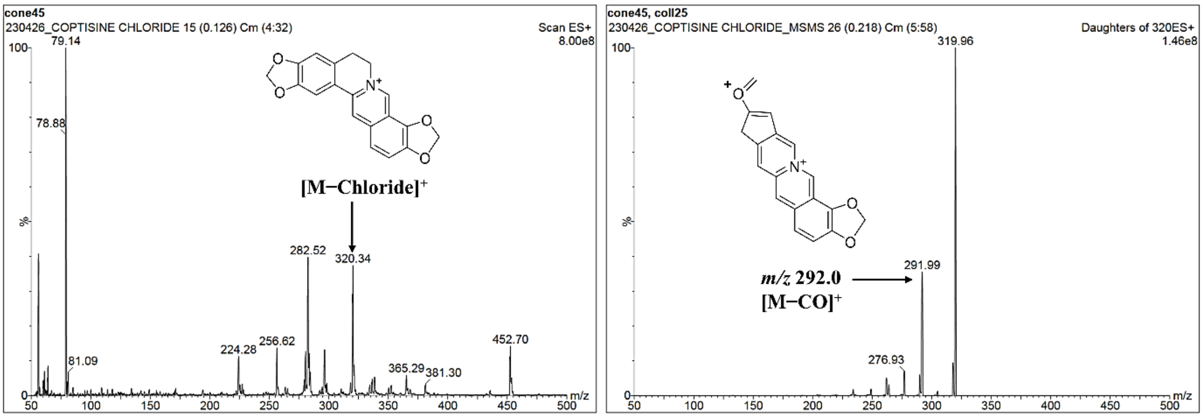

Tetrahydropalmatine

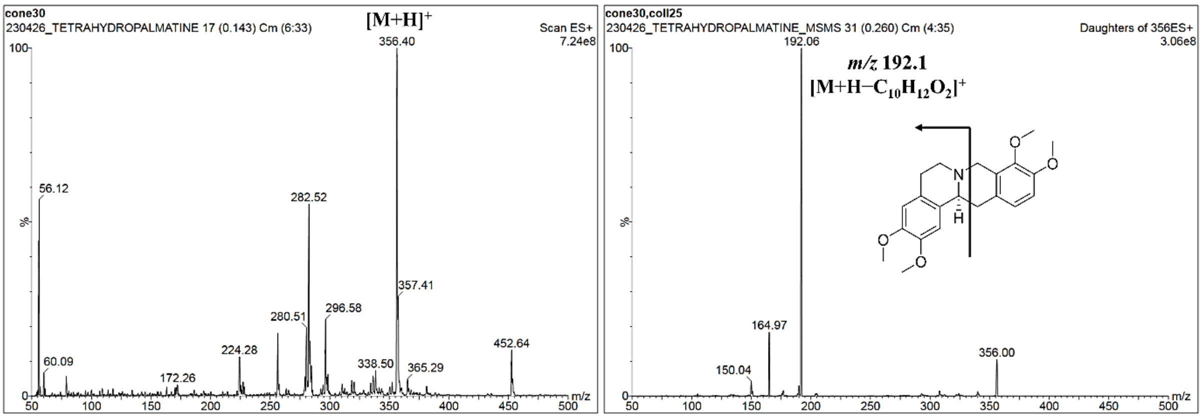

Tetrahydrocoptisine

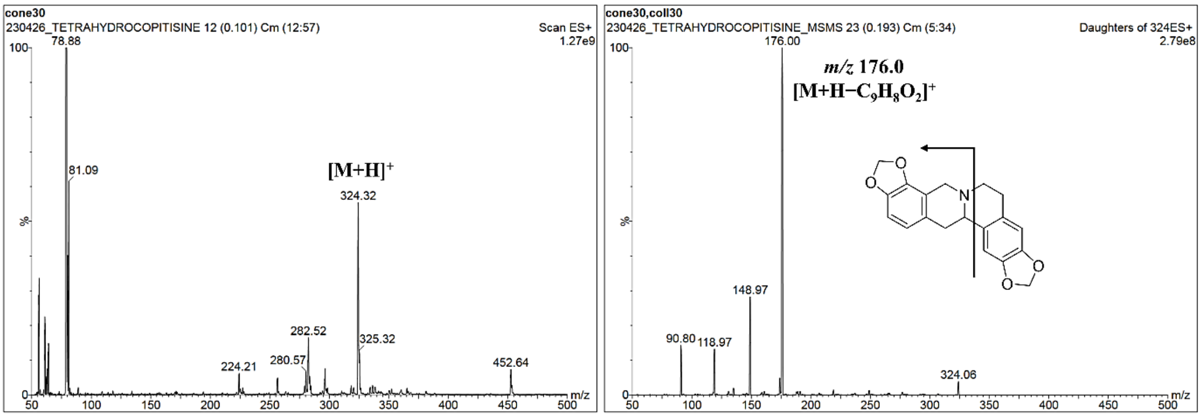

Berberrubine Cl

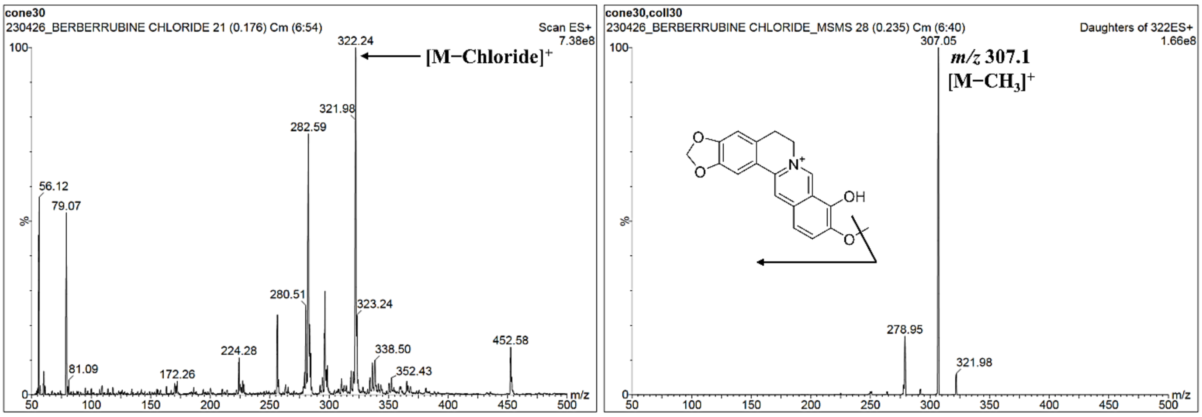

Canadine

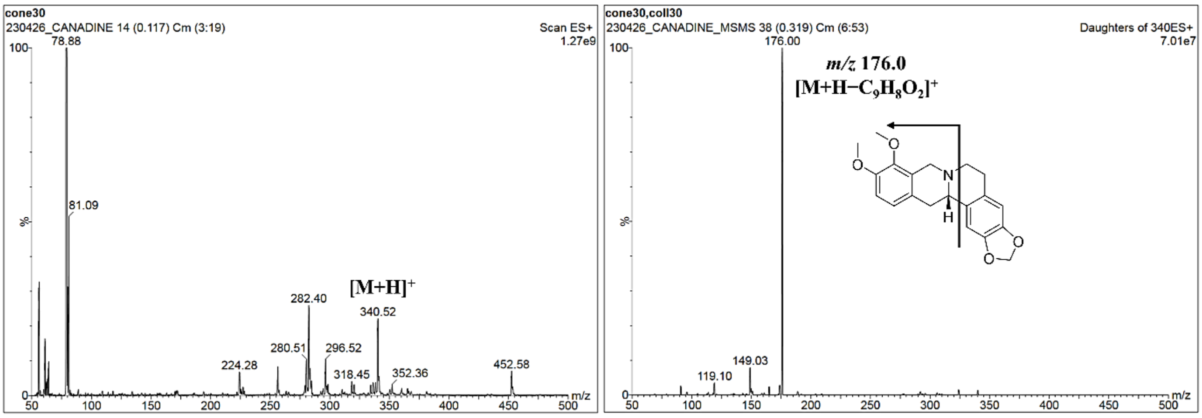

Corydaline

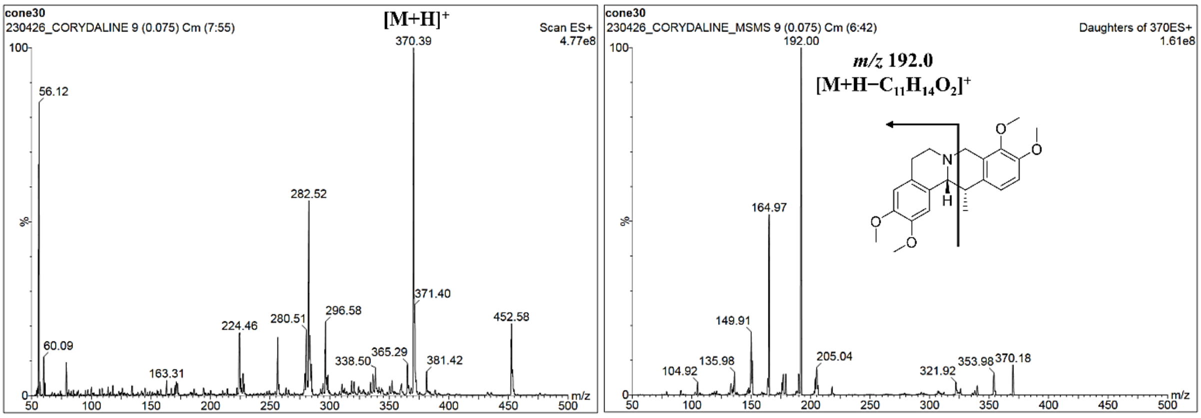

Palmatine Cl

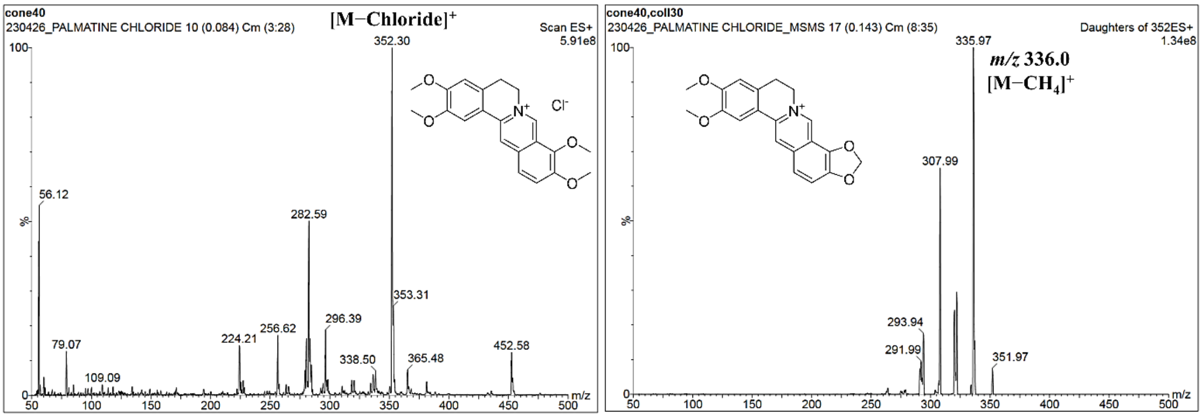

Berberine Cl

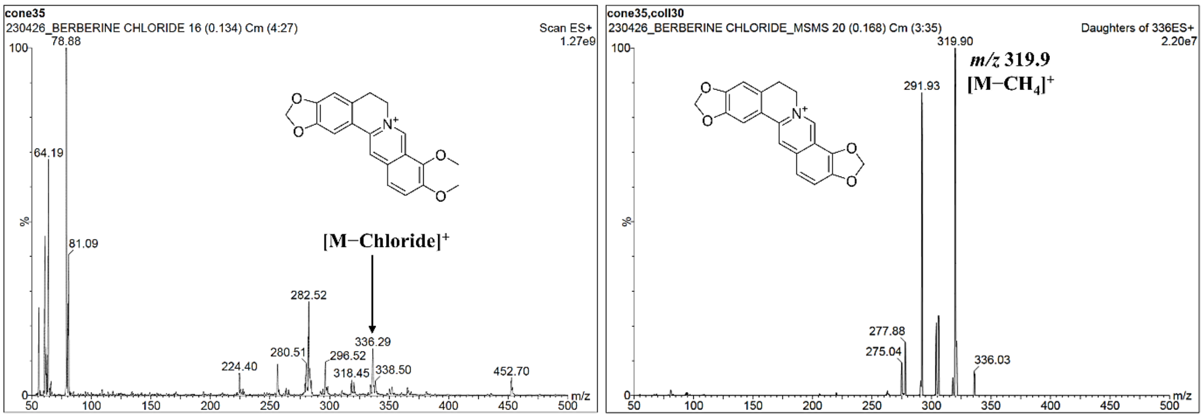

Dehydrocordaline

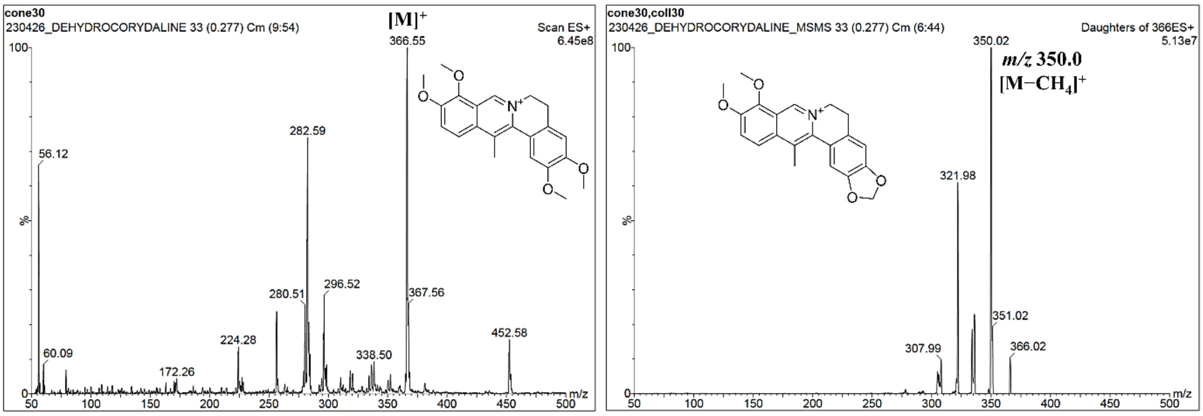

UPLC-MS/MS, ultra-performance liquid chromatography-tandem mass spectrometry.

Figure S2. Cytotoxicity of CTE and its compounds in HaCaT cells.

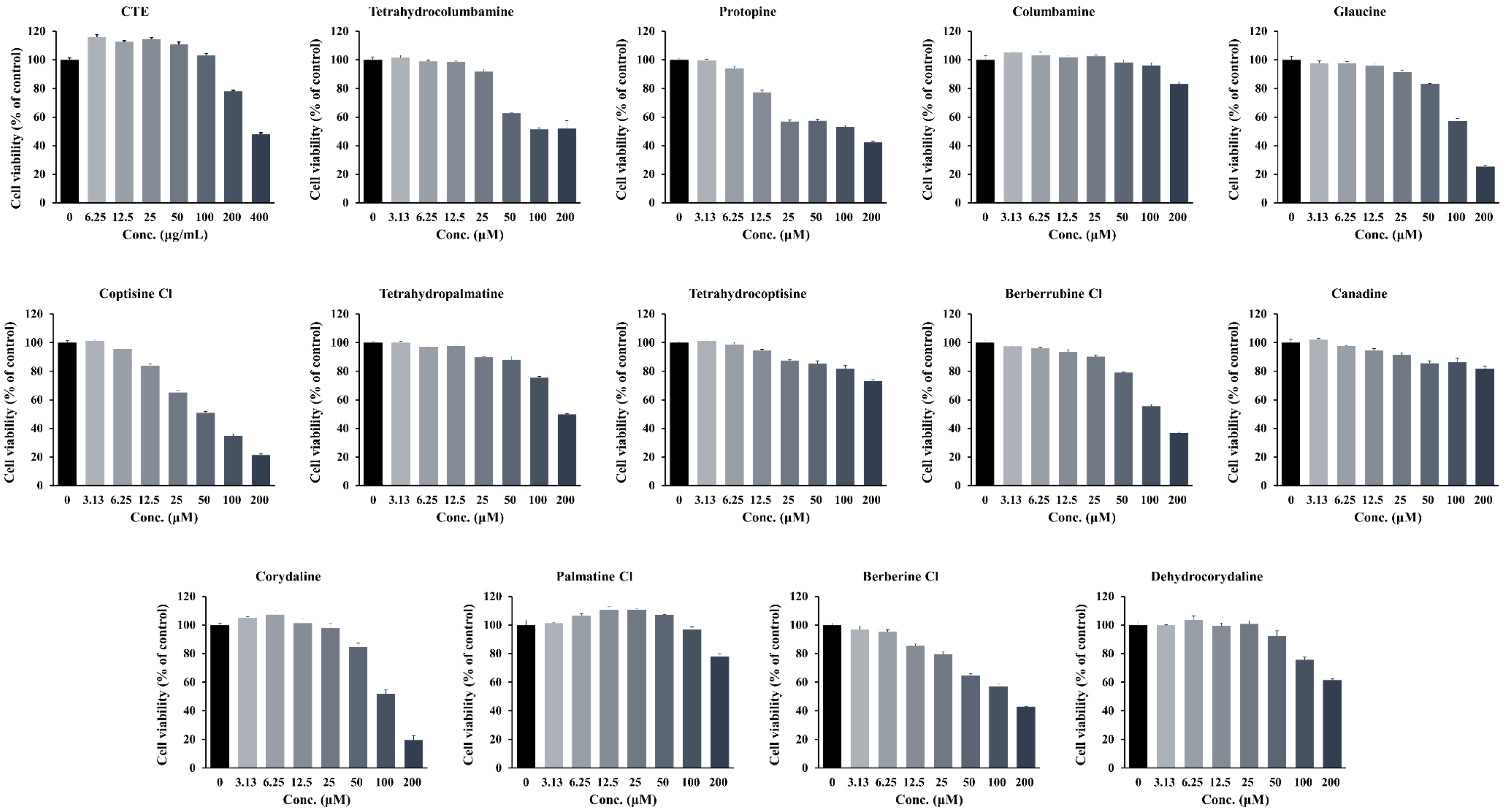

Data are expressed as means ± SEMs (n = 4). CTE, Corydalis Tuber 70% ethanol extract.

Figure S3. Effect of compounds included in CTE on chemokine production in TI-stimulated HaCaT cells.

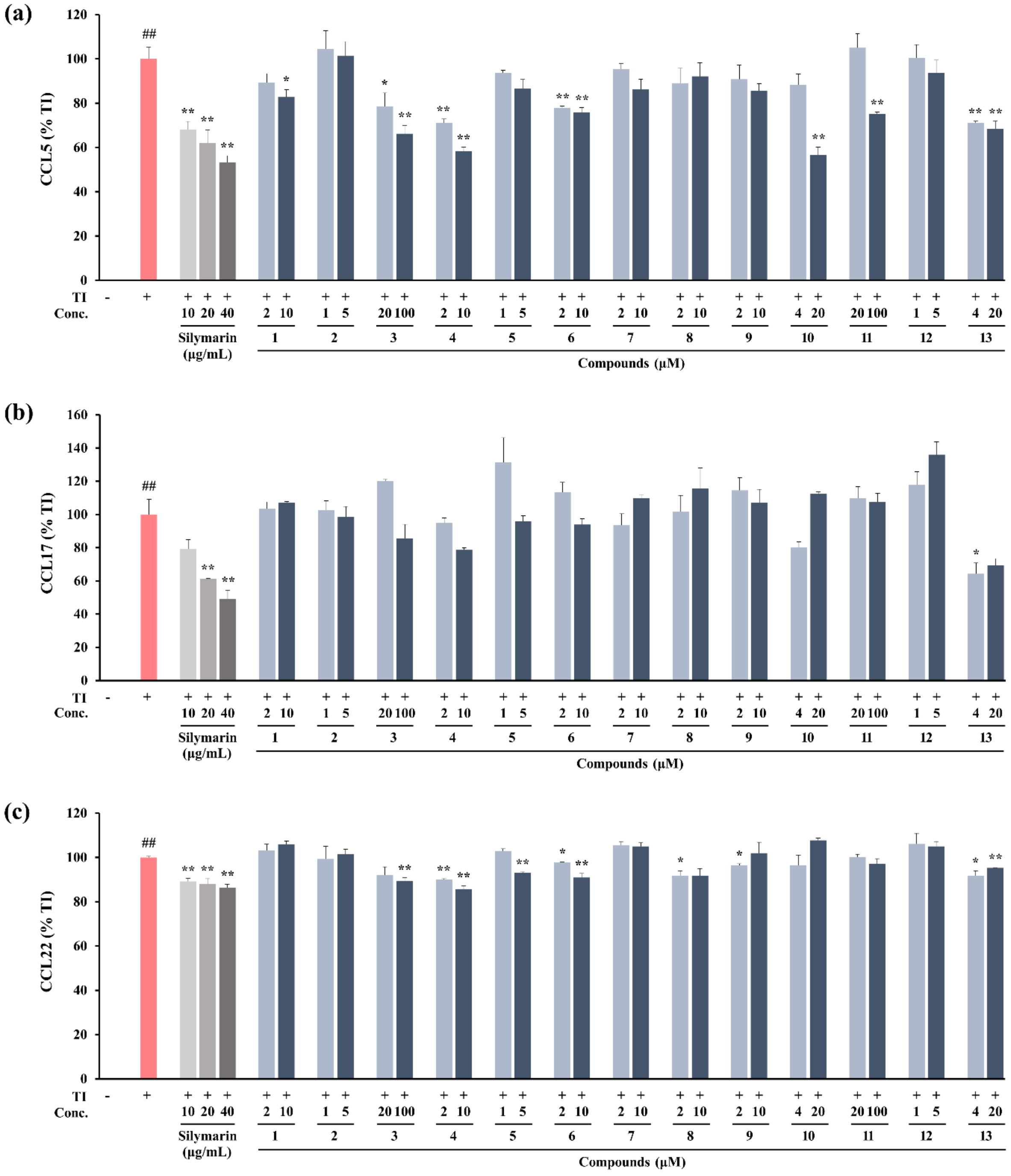

The cells were treated with CTE and stimulated with TI for 24 h. The levels of CCL5 **(a)**, CCL17 **(b)**, and CCL22 **(c)** in the supernatant were measured. Silymarin was used as a positive control. Data are expressed as means ± SEMs (n = 3). $^{\#\#}p < 0.01$ vs. normal control; $^{*}p < 0.05$ and $^{**}p < 0.01$ vs. TI-stimulated cells. CTE, Corydalis Tuber 70%

ethanol extract; TI, TNF-α (10 ng/mL) and IFN-γ (10 ng/mL); **1**, Tetrahydrocolumbamine; **2**, protopine; **3**, columbamine; **4**, glaucine; **5**, coptisine Cl; **6**, tetrahydropalmatine; **7**, tetrahydrocoptisine; **8**, berberrubine Cl; **9**, canadine; **10**, corydaline; **11**, palmatine Cl; **12**, berberine Cl; **13**, dehydrocorydaline.
